# Epidemiology, Detection, and Control of Foodborne Microbial Pathogens

**DOI:** 10.1155/2015/617417

**Published:** 2015-11-16

**Authors:** Miguel Prieto, Pierre Colin, Pablo Fernández-Escámez, Avelino Alvarez-Ordóñez

**Affiliations:** ^1^Veterinary Faculty, University of León, 24071 León, Spain; ^2^High School of Microbiology and Food Safety, University of Western Brittany, Technopôle Brest-Iroise, 29280 Plouzané, France; ^3^Higher Technical School of Agricultural Engineering, Polytechnic University of Cartagena, Cartagena, Spain; ^4^Teagasc Food Research Centre, Moorepark, Fermoy, County Cork, Ireland

Foodborne microbial diseases are a significant public health threat. They occur in both developed and developing countries with different food industry expansion, food safety regulations, food hygiene and consumption habits, and climate and environmental situations. The subsequent economic burden associated to them is also different. Most foodborne diseases are sporadic and often not reported, but sometimes foodborne outbreaks may affect a large number of individuals and compromise economic sectors and sanitary resources. Contamination of foods can occur at any point along the food chain, while pathogenic microorganisms enter the body through the ingestion of contaminated food.

A series of pathogens and diseases are emerging, driven by factors such as the change in pre- and postharvest stages of food production and manufacturing, pathogen adaptation and evolution, and changes in lifestyle, consumption habits, or host susceptibility, which modify the global exposure to foodborne pathogens. Other factors contemplated as possible contributors to the increased incidence include the recent changes in the food supply system, which result in intensive production and complexity of the supply chain. Because food production, manufacturing, and marketing are global, infectious agents present in foods can be disseminated from the original point of processing and packaging to the place of consumption thousands of kilometers away. Travel and expansion in international trade of foods have also increased the occurrence of outbreaks involving several countries and of cross-border transmission of agents and diseases.

There are a number of recent advances in the epidemiology, detection, and control of foodborne agents, including the development of novel methodologies and tools for the detection of pathogens, the growing availability of genome sequences which provide unrestricted information on bacterial genetics and physiology and give clues for the control of pathogenic microorganisms, the expanding knowledge on the epidemiology of the emerging pathogens, and the extensive application of molecular genomics and postgenomics tools for deciphering the behavior of foodborne pathogens in food-related environments. Surveillance studies contribute also to providing data and a better understanding into the existence and spread of foodborne pathogens. There is a special interest to know the origin and routes of contamination of foodborne pathogens, while farm animals and domestic and wild animals have been reported to be primary reservoirs for foodborne pathogens. It is also increasingly known that foodborne pathogens are able to change and adapt to different environmental conditions. The ability of these pathogens to develop adaptive response networks has contributed to their capacity to survive under a wide range of conditions and even stimulate their virulence potential.

This special issue comprises several reviews and original research articles which cover the most recent investigations on aspects such as the occurrence of foodborne pathogens, molecular typing of isolates, methods of detection or strain characterization aimed at foodborne pathogens, investigation and management of outbreaks, mathematical modelling of microbial growth and inactivation in foods, novel technologies of food preservation and microbial stress responses, and the impact of all this knowledge on food safety management, including the design of novel strategies for the (bio)control of foodborne pathogens.

Regarding occurrence surveys and molecular epidemiology studies, the contributions address a variety of relevant foodborne pathogens at international level in different animal and human populations and food categories (*Campylobacter jejuni* in poultry in Italy by F. Marotta et al.; Shiga toxigenic* Escherichia coli* O157 and non-O157 in ruminant feces in Malaysia by A. Perera et al.;* Salmonella enterica* in poultry and humans in Algeria by R. Elgroud et al.; enteropathogenic* Yersinia* spp. in pigs in Finland by M. J. Vilar et al.; food- and smear-transmitted pathogens [*E. coli*, norovirus,* Shigella* spp.,* Cryptosporidium parvum*,* Giardia duodenalis*,* Salmonella* spp., astrovirus,* Rotavirus*, and* Sapovirus*] in humans in Mali by H. Frickmann et al.;* Listeria ivanovii* in ready-to-eat foods and food processing environments in Ireland by A. Alvarez-Ordóñez et al.;* Listeria* spp. in food samples in Brazil by D. C. Vallim et al.;* Arcobacter butzleri* and* Arcobacter cryaerophilus* in pork, beef, and chicken meat in Poland by I. Zacharow et al.;* Escherichia coli* in conventional and free-range poultry in Brazil by V. L. Koga et al.;* Listeria monocytogenes* in hospital patients in Spain by J. Ariza-Miguel et al.).

A number of studies deal with methods of detection and isolation of foodborne pathogens and characterization of isolates. M. Srisawat and W. Panbangred describe a loop-mediated isothermal amplification method (LAMP) for the specific detection of* Salmonella* in food samples. A. Rohde et al. perform a systematic comparison of different homogenization approaches (stomaching, sonication, and milling) for the accurate detection of foodborne pathogens in meat. K. Jaakkola et al. report a comparative genomic hybridization analysis with a DNA microarray based on three* Yersinia enterocolitica* and four* Yersinia pseudotuberculosis* genomes to shed light on the genomic differences between enteropathogenic* Yersinia*. One research article by F. Sayk et al. describes the management activities implemented by the Emergency Department in relation to the German Shiga Toxin producing* Escherichia coli* (STEC) O104:H4 outbreak in two tertiary hospitals in Lubeck, northern Germany, in relation to patients with food-related diarrhoea.

Two studies are related to microbial growth in foods. C. M. McAuley et al. evaluate the growth of* Salmonella* Typhimurium and* Salmonella* Sofia on eggs under conventional production and retail conditions. M.-L. Pla et al. compare the accuracy of a variety of primary models to predict the growth of* Bacillus cereus*,* Listeria monocytogenes,* and* Escherichia coli* by the plate count and absorbance methods.

Several contributions focus on novel food preservation methodologies and food control strategies and on how microbial stress responses impact their efficacy. L. Wang and C. Shen assess the efficacy of hops beta acids (HBA) against unstressed and stress-adapted* Listeria monocytogenes* in ham extract and evaluate the consumers' acceptability of HBA on ready-to-eat ham. M. Gouma et al. establish the process criteria for using UV-C light alone or combined with mild heat (UV-H treatment) to inactivate 5 log_10 _cycles of* Escherichia coli*,* Salmonella* Typhimurium,* Listeria monocytogenes,* and* Staphylococcus aureus* in chicken broth. T. Hintz et al. review the use of plant-derived products as antimicrobial agents for food preservation to control foodborne pathogens. L. Guevara et al. assess the impact of moderate heat, combined with carvacrol and thymol on the viability, injury status, and stress response of* Listeria monocytogenes*. E. Jończyk-Matysiak et al. review the potential as control strategies of bacteriophages active against* Bacillus anthracis* and other* Bacillus cereus* group members.

This editorial summarizes the topics discussed in the articles published in this special issue, in the confidence that readers will find this information useful with the most recent research on major developments in the area of epidemiology, detection, and control of foodborne microbial pathogens.

